# Identification of LEA, a podocalyxin‐like glycoprotein, as a predictor for the progression of colorectal cancer

**DOI:** 10.1002/cam4.1765

**Published:** 2018-09-12

**Authors:** Dezheng Yuan, Hang Chen, Shuo Wang, Furong Liu, Yajie Cheng, Jin Fang

**Affiliations:** ^1^ Department of Cell Biology Key Laboratory of Cell Biology Ministry of Public Health Key Laboratory of Medical Cell Biology Ministry of Education China Medical University Shenyang China; ^2^ Analytical Instrumentation Center Shenyang Agricultural University Shenyang China

**Keywords:** CRC, exosome, LEA, ND‐1, PODXL

## Abstract

Large external antigen (LEA) is considered as a colorectal cancer (CRC)‐associated antigen, which was found via mAb ND‐1 generated using hybridoma technology, but its molecular features remain unknown. To facilitate the clinical application of LEA, we identified LEA as a podocalyxin‐like protein 1 (PODXL) with molecular weight of approximately 230 kDa, a hyperglycosylated protein, using immunoprecipitation and mass spectrometry in combination, and verified that ND‐1‐recognized epitope is on the terminal sialic acid of LEA. Correlation analysis between LEA and PODXL in molecular weight, immunological cross‐reactivity, and gene expression dependence supported the PODXL identity of the LEA. Moreover, we assessed the clinical significance of the LEA in 89 pairs of primary CRC tissues and adjacent nontumor colorectal tissues using ND‐1 by quantum dot‐based immunohistochemistry (QD‐IHC). High LEA expression was correlated significantly with T stage (*P *=* *0.010). Patients with high LEA expression showed significantly poorer prognosis than those with LEA low expression (*P *=* *0.007). Multivariate analysis indicated LEA expression as an independent predictor. Furthermore, the comparative analysis showed that mAb ND‐1‐based IHC analysis toward sugar residue of PODXL has higher sensitivity and specificity to evaluate the LEA/PODXL expression than mAb 3D3‐based method toward core protein of PODXL in CRC cell lines and clinical samples. In addition, we first found that LEA/PODXL can be secreted in exosomes from cancer cells and CRC patient peripheral blood. Our results demonstrate that LEA is an independent predictor for CRC progression and has the potential to be applied for clinical setting with high sensitivity, high specificity, and noninvasive access.

## 1 INTRODUCTION

Colorectal cancer (CRC) is the fifth most commonly diagnosed cancer in China.^1^ Approximately 25% of patients present with metastases at initial diagnosis, and nearly 50% of patients with CRC will develop metastases, contributing to the high mortality rate of the disease.^2^ Therefore, the early discovery of metastasis and prediction of recurrence are considerable critical to increase the survival rate of CRC patients. Currently, molecular detection based on biomarker changes is an effective strategy for cancer prediction prior to the appearance of detectable histopathological changes.^3^, ^4^, ^5^ Compared to other approaches, biomarker‐based detection is proven to have higher analytical sensitivity, which facilitates early diagnosis for various types of cancers.^6^, ^7^, ^8^ Furthermore, many biomarkers can be detected in the peripheral blood, leading to noninvasiveness and low‐cost superiority.^9^ For example, carcinoembryonic antigen (CEA) is a widely used biomarker with the ability to predict tumor recurrence in CRC and several other cancers.^10^, ^11^, ^12^, ^13^ According to a meta‐analysis summarized by Nicholson et al^14^, the sensitivity of CEA at threshold of 2.5 μg/L for detecting CRC recurrence reached to 82%.

Large external antigen (LEA) is considered as a CRC‐associated antigen that was discovered based on the specific recognition by ND‐1. ND‐1 is a mAb which was produced by hybridoma technology using the CRC CL187 cells as the immunogen.^15^ Previous researches showed that LEA was a glycoprotein mainly expressed on CRC cell membrane.^15^ IHC primarily revealed that LEA expression was associated with T stage of CRC.^16^ Notably, LEA was detectable in the serum and ascites of CRC patients, leading to a convenient clinical access.^15^ Accumulating data suggested that LEA might serve as a valuable diagnostic marker for CRC, but its molecular features remained unknown.

Identifying interest antigens by corresponding antibody is a widely used and effective strategy for exploring the molecular features of antigens and further discovering new biomarkers.^17^, ^18^, ^19^ CA19‐9, a commonly used serum‐based marker for monitoring pancreatic cancer recurrence, was identified as apolipoprotein B‐100 in pancreatic cancer by immunoprecipitation (IP) combined with mass spectrometry (MS) analysis.^20^ In this study, by enriching LEA using ND‐1‐based IP, followed by further analysis with LC‐MS/MS technology, we identified LEA as a podocalyxin‐like protein 1 (PODXL) with an apparent molecular weight of approximately 230 kDa.

PODXL is a transmembrane glycoprotein belonging to the CD34 family, which appears at various molecular weights in different tissues or cells due to changed glycoforms despite sharing a common core protein of approximately 55 kDa.^21^, ^22^ It was first discovered in renal podocytes as an antiadhesive protein with the molecular weight of 160‐165 kDa and later found to be expressed in vascular endothelium and involved in hematopoiesis and neural development.^23^, ^24^, ^25^, ^26^ Recently, aberrant PODXL expression was observed in several different types of cancers including breast cancer, bladder cancer, renal cancer, prostate cancer, pancreatic adenocarcinoma, and gastric adenocarcinoma.^27^, ^28^, ^29^, ^30^, ^31^, ^32^ Sizemore et al^30^ found that PODXL increased the aggressive phenotype of breast cancer and prostate cancer. In both pancreatic and periampullary adenocarcinoma and esophageal and gastric adenocarcinoma, PODXL was proven to be an independent predictor of poor outcome.^31^, ^32^ In the case of CRC, Larsson et al^33^ detected PODXL using antibody HPA2110 and analyzed its expression levels in 536 CRC cases, demonstrating that the PODXL was an independent factor of poor prognosis and may be a useful marker to stratify patients for adjuvant chemotherapy.

To further investigate the clinical significance of LEA, a PODXL protein recognized by ND‐1, we used QD‐IHC assay to determine LEA expression in 89 CRC samples and found that LEA expression was related to T stage, and LEA might have potential as an independent predictor of the poor prognosis for CRC patients. Furthermore, ND‐1 showed superior sensitivity to the commercial mAb 3D3 against PODXL core protein when used to analyze LEA expression in CRC samples. Moreover, we first verified that LEA could be secreted in exosomes from cancer cells and CRC patient peripheral blood, which provides more convenient access for understanding the function of LEA in metastasis as well as its significance in clinical settings.

## 2 MATERIALS AND METHODS

### 2.1 Cell lines and clinical specimens

The human CRC cell lines Colo205, LS174T, SW480, and human embryonic kidney cell line HEK293 were obtained from the Type Culture Collection of the Chinese Academy of Sciences (Shanghai, China). The human CRC cell lines CL187 and HT29 were purchased from American Type Culture Collection (Manassas, VA, USA). All culture media were supplemented with 10% FBS and 100 units/mL penicillin‐streptomycin. The cultures were maintained in a humidified atmosphere containing 5% CO_2_ at 37°C.

Two commercial human CRC tissue microarrays (TMAs) were purchased from Shanghai Outdo Biotech, China. One TMA (catalogue no. HCol‐Ade180 Sur‐07) contained 90 pairs of tissues (primary CRC cancer tissues and adjacent nontumor colorectal tissues for each pair). All patients had undergone curative resection from January to October 2009; the last follow‐up occurred in July 2015. The other TMA (catalogue no. HCol‐Ade060CS‐01) consisted of 28 cases of CRC tissues, including 21 nonmetastatic, seven metastatic, and four normal colorectal tissues. The clinicopathological characteristics of the samples are shown in Table 1.

**Table 1:** Association between clinicopathological characteristics and LEA expression

	LEA expression			χ^2^	Spearman's correlation	
n (%)	Negative 5 (5.6)	Weak‐moderate 60 (67.4)	Strong 24 (27.0)	*P*‐value	*r*	*P*‐value
Age				0.571	‐0.054	0.616
≤60	2 (40.0)	17 (28.3)	9 (37.5)			
>60	3 (60.0)	43 (71.7)	15 (62.5)			
Gender				0.620	‐0.043	0.692
Female	3 (60.0)	27 (45.0)	13 (54.2)			
Male	2 (40.0)	33 (55.0)	11 (45.8)			
Tumor size (cm)				0.671	‐0.035	0.749
≤5	1 (20.0)	27 (45.0)	10 (41.7)			
>5	4 (80.0)	33 (55.0)	14 (58.3)			
Histology				1.000	0.053	0.622
Adenocarcinoma	5 (100.0)	53 (88.3)	21 (87.5)			
Mucinous carcinoma	0	5 (8.4)	2 (8.3)			
Others	0	2 (3.3)	1 (4.2)			
T stage				**0.012** a	**0.270**	**0.010** a
T_1‐2_	3 (60.0)	9 (15.0)	1 (4.2)			
T_3‐4_	2 (40.0)	51 (85.0)	23 (95.8)			
N stage				0.931	0.021	0.847
N_0_	3 (60.0)	40 (66.7)	15 (62.5)			
N_1‐2_	2 (40.0)	20 (33.3)	9 (37.5)			
M stage				1.000	0.040	0.711
M_0_	5 (100.0)	58 (96.7)	23 (95.8)			
M_1_	0	2 (3.3)	1 (4.2)			

a *P* < 0.05 indicates a statistically significant difference.

The plasma samples from CRC patients and healthy donors for exosome detection were obtained from the First Affiliated Hospital of China Medical University. The study was approved by the ethics review committees at The First Affiliated Hospital of China Medical University.

### 2.2 Antibodies and plasmid

ND‐1, the mAb of LEA, was obtained and purified as described previously.^16^ Mouse anti‐PODXL mAb (3D3, cat#sc‐23904) and normal mouse IgG (cat#sc‐2025) were purchased from Santa Cruz Biotechnology (Dallas, TX, USA). Rabbit anti‐TSG101 mAb (cat#ab30871) was obtained from Abcam (Cambridge Science Park, Milton, Cambridge, UK). Mouse anti‐CD63 mAb (cat#10628D) was purchased from Invitrogen Corporation (Carlsbad, CA, USA). Mouse anti‐actin mAb (TA‐09), mouse anti‐GAPDH mAb (TA‐08), sheep anti‐mouse FITC‐IgG (ZF‐0315), sheep anti‐mouse IgG‐HRP (ZDR‐5307), and sheep anti‐rabbit IgG‐HRP (ZDR‐5306) were obtained from ZSGB‐BIO (Beijing, China). The plasmid pcDNA3.1‐PODXL was a generous gift from Dr. Ayuso MS, CSIC, Madrid, Spain.

### 2.3 Immunofluorescence assay

CL187 cells (1 × 10^6^ cells/mL) were first incubated with primary antibodies ND‐1 (1:1000 dilution) or 3D3 (1:200 dilution) followed by incubation with a FITC‐conjugated anti‐mouse secondary antibody (1:500 dilution). Subsequently, the cells were incubated with DAPI (cat#32670; Sigma‐Aldrich, Poznań, Poland) and the images were obtained under a confocal microscope (Olympus, Tokyo, Japan).

### 2.4 Western blotting and IP

Cells were lysed in RIPA lysis buffer. Proteins were separated by SDS‐PAGE and transferred onto a PVDF membrane. After being blocked with 5% BSA, the membranes were incubated with various primary antibodies including ND‐1 (1:3000 dilution), 3D3 (1:500 dilution), anti‐TSG101 (1:1000 dilution), anti‐CD63 (1:500 dilution), anti‐actin (1:3000 dilution), or anti‐GAPDH (1:3000 dilution) at 4°C overnight and subsequently probed with corresponding horseradish peroxidase‐conjugated secondary antibodies at RT for 1 hour. The protein bands were developed using a chemiluminescence imaging analysis system. GAPDH or β‐actin was used as a loading control.

CL187 cells were chosen for IP assay to identify the immunological cross‐reactivity between LEA and PODXL. Total protein was collected from CL187 cells and precleaned with Protein G PLUS‐Agarose beads, then incubated with ND‐1 or 3D3 at 4°C overnight, followed by incubation with Protein G PLUS‐Agarose beads at 4°C for 2 hours. Beads were collected after washing with IP buffer, followed by the elution with the sample buffer. The resultant proteins were subjected to SDS‐PAGE and analyzed by western blotting using appropriate antibodies as described above.

### 2.5 Gel‐based mass spectrometry analysis

The ND‐1‐immunoprecipitates from CL187 cells lysate were analyzed by SDS‐PAGE and western blotting, respectively. The band of interest on SDS‐PAGE gel was excised and subjected to trypsin digestion, followed by peptide separation and MS analysis on an LTQ Orbitrap Velos mass spectrometer (Thermo Fisher Scientific, Carlsbad, CA) by Applied Protein Technology (Shanghai, China). The results were analyzed using the Bioworks Browser 3.1 software (Thermo Finnigan, Thermo Electron Corporation, Austin, TX, USA).

### 2.6 Silence and overexpression of PODXL gene

Small interfering RNAs (siRNAs) targeting human PODXL gene were designed and synthesized by Applygen, Technology Inc. (Beijing, China). The sequences were as following: PODXL1478, 5′‐GUGGUCGUCAAAGAAAUCATT‐3′; PODXL867, 5′‐CGGGACAUGACCAUCUUAUTT‐3′; PODXL671, 5′‐GCCACAGCUAAACCUAACATT‐3′; negative control, 5′‐UUCUCCGAACGUGUCACGUTT‐3′. CL187 cells were plated in six‐well plates (1 × 10^5^ cells/well) and transfected with siRNAs by Higene (Applygen, Beijing, China) according to the manufacturer's protocol. After 48 hours transfection, protein expression was analyzed by western blotting using 3D3 and ND‐1 as primary antibodies. For overexpression, HEK293 cells (1 × 10^5^ cells/well) were transfected with pcDNA3.1‐PODXL and the expression of LEA or PODXL were analyzed by western blotting as described above.

### 2.7 Analysis of LEA glycosylation form

To identify the glycosylation form of the LEA molecule, Colo205 cells (1 × 10^6^ cells/mL) were treated with benzyl‐2‐acetamid‐o‐2‐deoxy‐α‐d‐galactopyranoside (BG) (cat#B4894; Sigma‐Aldrich, St Louis, MO, USA), an inhibitor of *O*‐linked glycosylation, in a series of concentrations (0, 0.25, 0.5, and 1.0 mmol/L). After 24 hours of incubation at 37°C, proteins were analyzed by western blotting using ND‐1 or 3D3 as primary antibodies.

### 2.8 Analysis of the sugar residue feature of LEA by sialidase treatment

To verify the characteristics of the sugar residues of LEA, a sialic acid‐specific hydrolytic enzyme reaction was conducted. CL187 cells (1 × 10^6^ cells/mL) were incubated with sialidase (cat#N2876; Sigma‐Aldrich, St Louis, MO, USA) at concentration of 1 unit/mL in HBSS at 37°C for 5 hours. Subsequently, the cells were incubated with ND‐1 (1:100 dilution) at RT for 2 hours, followed by the incubation with FITC‐conjugated anti‐IgG secondary antibody (1:500 dilution) at RT for 1 hour in the dark. Cells were then subjected to flow cytometry (Becton‐Dickinson, San Jose, CA, USA).

### 2.9 QD‐IHC

Human CRC TMA was subjected to QD‐IHC assay as described previously.^16^ The fluorescent images were obtained by fluorescence microscope (Carl Zeiss, Oberkochen, Germany), then the fluorescence intensity of images was read by MetaMorph software (UIC, Downingtown, PA, USA) and scored. Three randomly selected fields for each section were imaged and measured, and average intensity was used for analysis. LEA/PODXL expression was scored as follows: 0 for negative expression, 1+ for weak expression, 2+ for moderate expression, and 3+ for strong expression.

### 2.10 Preparation, characterization, and LEA expression analysis of exosomes

Exosomes in culture medium of CL187 cells were isolated by differential centrifugation. Briefly, cells were grown in high‐glucose DMEM supplemented with 10% exosome‐depleted FBS. After 48 hours, culture medium was collected and subjected to sequential centrifugation at 300 *g* for 5 minutes and 15,000 *g* for 15 minutes at 4°C to remove residual cells and debris. After filtered with a 0.22 μm Millex‐GV filter (Millipore, Billerica, MA, USA), the resultant supernatant was ultracentrifuged at 150,000 g for 3 hours at 4°C, and the exosome pellets were resuspended in PBS for use. For the analysis of plasma‐derived exosomes, peripheral blood samples (4 mL each) were collected in anticoagulant tubes from healthy donors or CRC patients, and the supernatant was obtained by centrifugation at 2000 *g* for 10 minutes. The exosomes were isolated by ultracentrifugation as above.

The morphologic features of exosomes were characterized by negative staining electron microscopy. The images were taken by a transmission electron microscope (HT7700 Hitachi microscope, Tokyo, Japan) at 100 kV. Two exosome markers, TSG101 and CD63, and LEA were detected by western blotting.

### 2.11 Statistical analysis

Associations between LEA expression and clinicopathological characteristics were analyzed by Spearman's correlation analysis and the chi‐square test. Patient's overall survival (OS) was analyzed with log‐rank test and Kaplan‐Meier analysis. Additionally, univariate and multivariate Cox‐regression analyses were used to determine the hazard ratio considering the LEA expression levels and subjects’ characteristics. *P *<* *0.05 (two‐sided) were considered significant. All statistical analysis was performed with IBM SPSS 20.0 (IBM Corporation, Armonk, NY, USA).

## 3 RESULTS

### 3.1 Identification of LEA

**Figure 1:**
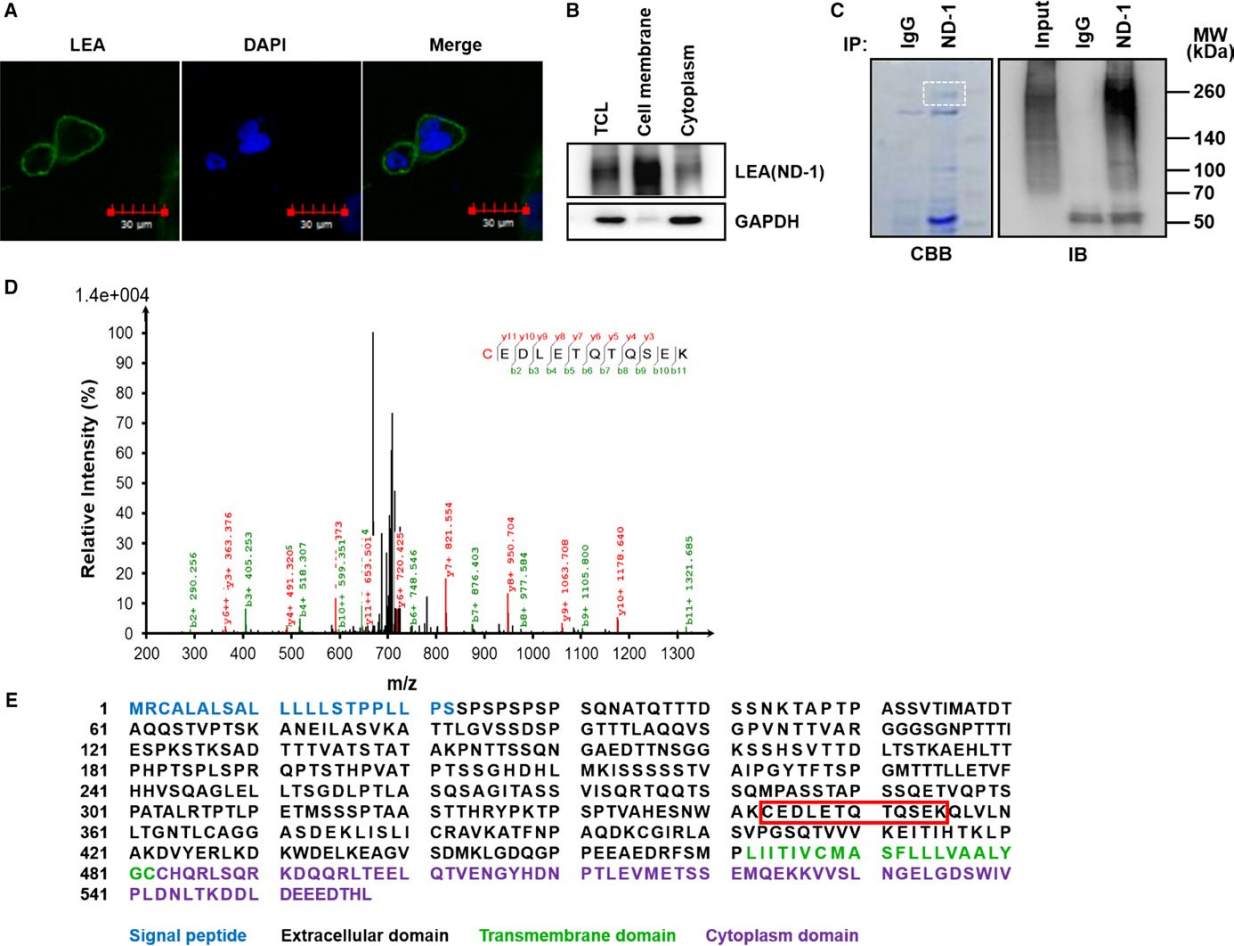
Identification of LEA as a PODXL protein in CL187 cells. A, Analyzing the cellular localization of LEA by immunofluorescence method with FITC‐labeled ND‐1. Scale bars: 30 μm. B, Analyzing the cellular distribution of LEA by western blotting. GAPDH was used as internal control of cytoplasmic protein. C, Analysis of the LEA immunoprecipitated by ND‐1 using SDS‐PAGE (left) and western blotting (right). The band of interest in white dashed box is cut off for MS analysis. D, The ion fragment spectrum of ND‐1‐immunoprecipitates by LC‐MS/MS analysis. E, Primary structure of PODXL. The peptide sequence identified by MS is shown in red box. TCL, total cell lysate; CBB, coomassie brilliant blue staining; IB, immunoblotting; MW, molecular weight

To examine LEA localization, CL187 cells were subjected to immunofluorescence assay by FITC‐labeled ND‐1. As shown in Figure 1A, an obvious green fluorescence was observed on the surface of CL187 cells, suggesting that LEA might be a membrane protein. Further, western blotting assay was performed to analyze LEA expression in CL187 cells using ND‐1. Data showed that LEA was mainly expressed in the cell membrane fraction, with an apparent molecular weight of approximately 230 kDa (Figure 1B).

To specifically identify LEA, LC‐MS/MS analysis was performed. LEA was first enriched from CL187 cells using ND‐1 by IP. The IP products were resolved by SDS‐PAGE and western blotting. The band at 230 kDa probed by ND‐1 was excised, in‐gel digested with trypsin and subjected to LC‐MS/MS analysis (Figure 1C). As shown in Figure 1D,E, a peptide fragment with the sequence of CEDLETQTQSEK matched amino acid residues 342‐355 of podocalyxin‐like protein 1, a transmembrane glycoprotein, which possesses the molecular weight of above 200 kDa in some cases.^34^, ^35^ These results demonstrated that LEA might be the PODXL protein.

**Figure 2:**
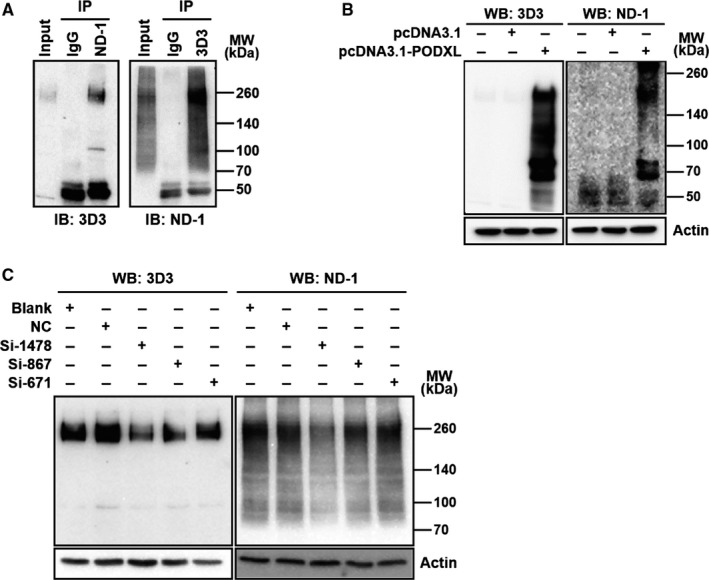
Verification of LEA as PODXL. A, Immunological cross‐reactivity between LEA and PODXL in CL187 cells was analyzed by IP assay with ND‐1 (left) and 3D3 (right), respectively, and then cross‐detected by western blotting with 3D3 (left) and ND‐1 (right). LEA expression depended on PODXL gene was validated using an overexpression assay in HEK293 cells (B), and siRNA assay in CL187 cells (C). IB, immunoblotting; WB, western blotting; MW, molecular weight

To verify the PODXL identity of the LEA, the immunological relationship of PODXL and LEA was studied. First, the same localization and the similar electrophoretic migration rate of LEA and PODXL in CL187 cells were shown in Figure S1A and B. Then, the immunological cross‐reactivity of LEA and PODXL was detected by IP assay. Lysates of CL187 cells were immunoprecipitated with ND‐1 (or 3D3), and the immunoprecipitates were cross‐detected using 3D3 (or ND‐1) by western blotting. As shown in Figure 2A, LEA and PODXL could be cross‐recognized by mAb of each other, indicating that LEA and PODXL belonged to the same protein. The distinct band patterns of LEA and PODXL suggested that they had different antigen epitopes.

**Figure 3:**
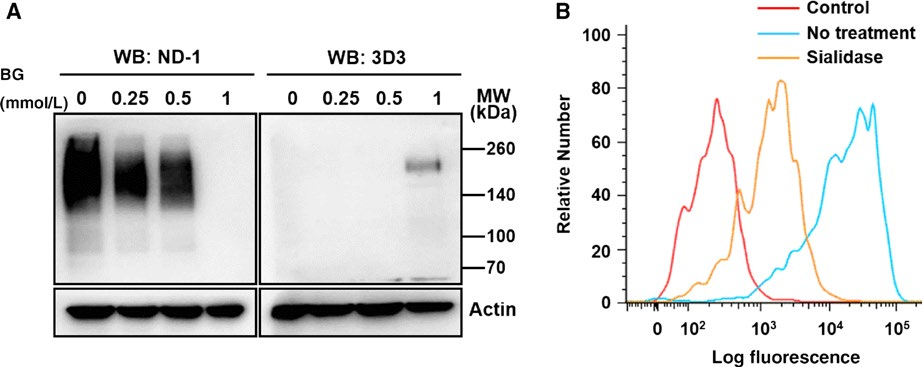
Analysis of the glycosylation feature of PODXL and LEA. A, *O*‐glycosylation inhibition assay. CL187 cells were treated by BG and then subjected to western blotting. B, Sialic acid digestion assay. CL187 cells were treated with sialidase and then analyzed by flow cytometry assay with FITC‐labeled ND‐1. WB, western blotting; MW, molecular weight

Next, we analyzed whether LEA detection was dependent on PODXL gene expression. HEK293 cells, which was lack of endogenous PODXL expression,^36^ were transfected with pcDNA3.1‐PODXL. Western blotting showed that ND‐1 could detect exogenous PODXL expression (Figure 2B). Further, PODXL expression was silenced in CL187 cells. As shown in Figure 2C, the results showed that a consistent decrease was found by western blotting analysis using ND‐1 and 3D3, indicating that down‐regulated LEA expression was dependent on PODXL gene silence. All these results indicated that LEA expression level depends on the PODXL gene expression and suggested that LEA and PODXL share the same gene, PODXL gene. PODXL has been reported to be an extensively *O*‐linked glycosylated glycoprotein.^21^ To verify whether LEA possesses the same glycosylation type, the glycosylation inhibitors were introduced to the study. Total lysates of BG (*O*‐linked glycosylation inhibitor) treated Colo205 cells were subjected to SDS‐PAGE followed with western blotting using antibodies ND‐1 and 3D3. As shown in Figure 3A, LEA level detected by ND‐1 gradually decreased along with the increase in BG concentration compared to control cells and finally disappeared at a concentration of 1 mmol/L, indicating that LEA is an *O*‐linked glycoprotein and that its ND‐1‐recognized epitope is on the *O*‐linked carbohydrates. Similarly, when detected by 3D3, detectable PODXL was impacted by BG but at a relatively high concentrantion of 1 mmol/L, suggesting that PODXL is an *O*‐linked glycoprotein, and further indicating the PODXL identity of the LEA. It was reported that the terminal sugar residue of PODXL is sialic acid,^37^ to confirm this, CL187 cells treated with sialidase were subjected to flow cytometry assay. As shown in Figure 3B, sialidase treatment induced a significant left shift of the fluorescence peak, indicating that LEA possessed the same terminal sugar residue as PODXL and the recognition epitope of ND‐1 was localized on the terminal sialic acid of PODXL.

### 3.2 LEA expression in CRC specimen

**Figure 4:**
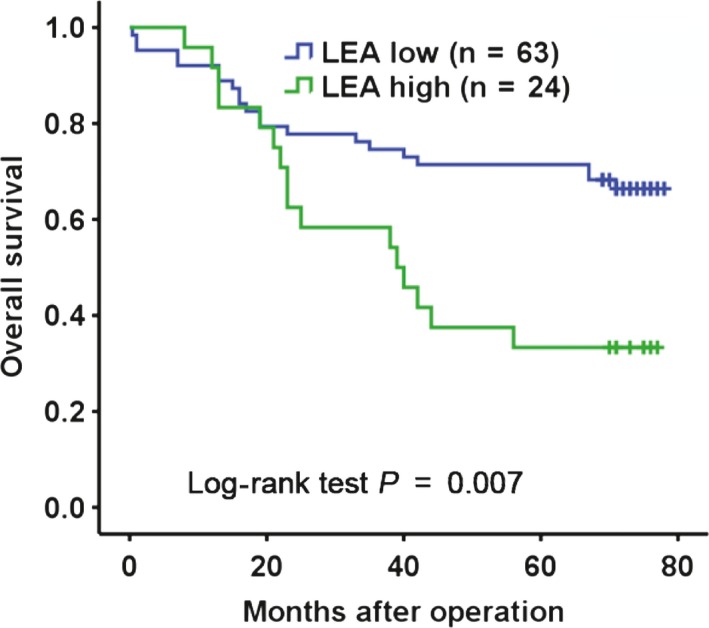
Kaplan‐Meier analysis of the association between LEA/PODXL expressions and OS of CRC patients. LEA low: LEA negative/low/moderate expression; LEA high: LEA strong expression

Based on above results, to investigate the clinical signification of LEA, the PODXL recognized by ND‐1, we analyzed LEA expression in a TMA containing 89 CRC specimens and adjacent nontumor colorectal tissues by QD‐IHC method. LEA expression levels were quantitatively classified into four grades 0, 1+, 2+, and 3+ according to its intensity of fluorescence (Figure S2). Data revealed that LEA expression was positively associated with T stage (*r *=* *0.270, *P *=* *0.010) and was not associated with age, sex, tumor size, histology, N stage, or M stage (Table 1, 89 cases available). Combined with Kaplan‐Meier analysis and log‐rank test, LEA expression was significantly relevant to OS of CRC patients (Figure 4, 87 cases of follow‐up data available), suggesting that CRC patients with LEA high expression had a lower OS than those with LEA low expression. Moreover, univariate and multivariate analyses both showed that LEA expression was an independent prognostic indicator for OS of CRC patients (Table 2).

**Table 2:** Uni‐ and multivariate analyses of the OS in 87 CRC patients

Clinicopathological characteristics	Hazard ratio (95% CI)	*P*‐value	Hazard ratio (95% CI)	*P*‐value
	Univariate		Multivariate	
Gender (female vs male)	1.414 (0.733‐2.727)	0.301	1.314 (0.640‐2.701)	0.457
Age (≤60 vs >60 y)	0.993 (0.499‐1.977)	0.984	0.778 (0.377‐1.606)	0.498
Tumor size (5≤ vs >5 cm)	2.249 (1.109‐4.560)	**0.025** a	2.332 (1.066‐5.103)	**0.034** a
Histology (adenocarcinoma vs mucinous carcinoma vs others)	0.650 (0.156‐2.711)	0.554	0.679 (0.158‐2.916)	0.603
	1.665 (0.399‐6.952)	0.485	3.789 (0.728‐19.729)	0.114
Tumor invasive depth (T_1‐2_ vs T_3‐4_)	1.488 (0.527‐4.201)	0.453	0.572 (0.168‐1.948)	0.372
Lymph node metastasis (N_0_ vs N_1‐2_)	3.099 (1.617‐5.940)	**0.001** a	3.653 (1.769‐7.543)	**<0.001** a
Distant organs metastasis (M_0_ vs M_1_)	5.622 (1.632‐19.372)	**0.006** a	6.624 (1.748‐25.102)	**0.005** a
LEA expression (low vs high expression)	2.389 (1.241‐4.601)	**0.009** a	2.233 (1.125‐4.432)	**0.022** a

CI: confidence interval.

a *P *<* *0.05 indicates that the lower limit of the 95% CI of hazard ratio is >1.

### 3.3 The application potential of LEA in clinical settings

**Figure 5:**
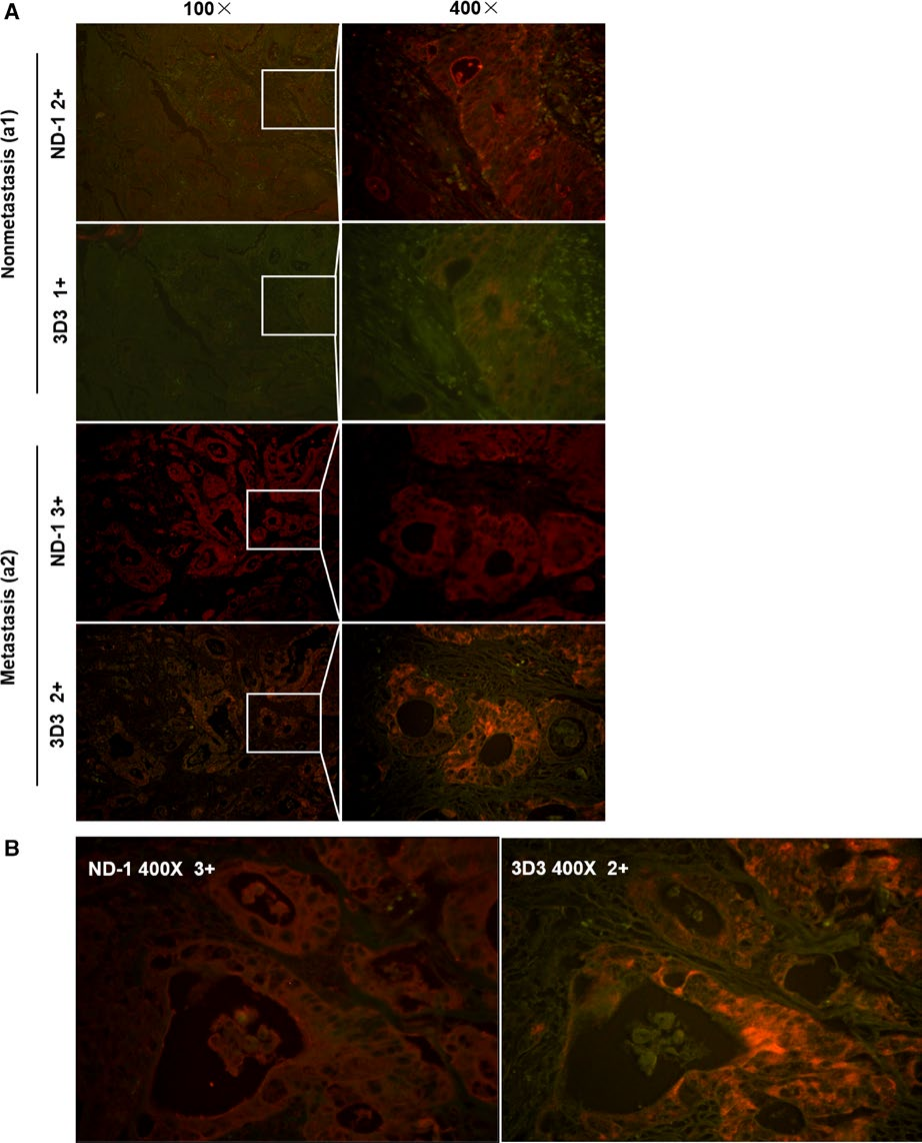
Comparative analysis of LEA and PODXL expression levels in CRC TMAs by QD‐IHC using mAb ND‐1 and 3D3, respectively. Representative images (×100, ×400, magnification) of nonmetastatic tissues (Aa1), metastatic tissues (Aa2), and tissues with glandular cavity expression (B)

We demonstrated that mAb ND‐1 recognized the sialic acid residue of LEA, which is different from a commonly used commercial mAb 3D3 against the core protein of PODXL. To investigate whether ND‐1‐based LEA detection has a better potential for clinical application, the LEA expression detected by ND‐1 was compared with that by 3D3 in CRC cell lines and in TMAs. First, protein levels in five CRC cell lines were analyzed by western blotting. As shown in Figure S3, by using 3D3, all of the cell lines except for Colo205 were detectable, while by using ND‐1, LEA could be detected in CL187, LS174T, and Colo205 cells, most of which were derived from metastatic tumors. Further, a TMA containing 28 CRC specimens with distinct metastatic feature was evaluated by QD‐IHC. Although the positive rates of LEA expression detected by 3D3 and ND‐1 were similar (92.86% vs 96.43%), the percentage of the strong positive expression (3+ group) by ND‐1 was significantly higher than that by 3D3 (46.43% vs 10.71%), especially in metastatic specimens. The representative results were shown in Figure 5.

**Figure 6:**
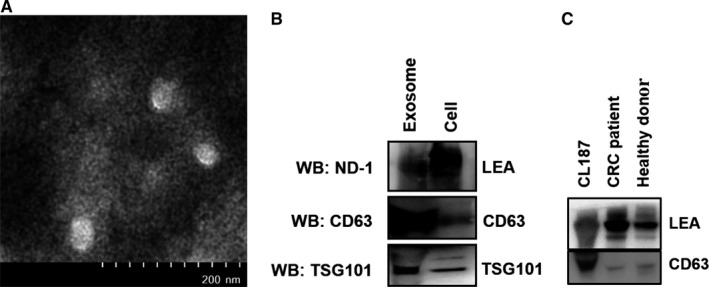
Characterization of exosomes derived from CL187 cells and CRC patient plasma. A, Representative electron microscope images of exosomes isolated from CL187 cells. B, Western blotting analysis of LEA and exosome‐marker expression in exosomes from CL187 cells using ND‐1, anti‐TSG101, and anti‐CD63, respectively. C, Representative analysis of LEA expression in exosomes from CRC patient and healthy donor plasma by western blotting analysis using ND‐1

Previous studies have shown that LEA could be secreted into extracellular space, and the present study also revealed a high LEA expression in the glandular cavity of CRC tissues (Figure 5B). We therefore investigated whether LEA exists in exosome form. First, exosomes were collected from the culture medium of CL187 cells and characterized by electron microscopy and western blotting. As shown in Figure 6A, the exosomes displayed a typical cup or round shape with a size of approximately 30 to 50 nm. Proteins in exosomes were subjected to western blotting and LEA showed a positive result together with TSG101 and CD63, the two markers for exosome (Figure 6B), which indicated that LEA might be secreted via exosomes. Further, the plasma‐derived exosomes from the CRC patients and healthy donors were isolated and detected. Western blotting analysis showed that the plasma‐derived exosomes could express LEA/PODXL, and two of eight CRC patient samples displayed higher LEA/PODXL expression than all six of healthy donor samples did. The representative results were shown in Figure 6C.

## 4 DISCUSSION

To identify the molecular features of LEA, we first enriched LEA from CL187 cells and then performed LC‐MS/MS analysis. Based on the characters of LEA as a membrane glycoprotein and its molecular weight, we inferred that LEA is PODXL, which contained an amino acid sequence matching for the LEA fragment detected by MS. Furthermore, immunofluorescence and western blotting assays confirmed that LEA had consistent membrane localization and molecular weight with PODXL in CL187 cells. IP revealed that LEA and PODXL could be cross‐recognized by antibodies of each other; PODXL gene overexpression and silence assays demonstrated that LEA expression was strongly dependent on PODXL gene. Glycosylation inhibition assay showed that LEA and PODXL were modified consistently by *O*‐linked sugar residues. All these results supported that identity of LEA recognized by ND‐1 was PODXL.

Previous studies have shown that LEA expression level is related to T stage of CRC patients.^16^ To further verify the clinical significance of PODXL recognized by ND‐1 in CRC, we detected LEA expression level in TMA, which contains 89 CRC tissues and adjacent nontumor colorectal tissues, by QD‐based IHC with mAb ND‐1. The positive expression of LEA in CRC tissues (94.4%) was much higher than that in nontumor colorectal tissues (8.9%), revealing a strong cancer‐specific expression. Clinical analysis revealed an obvious positive correlation between high LEA expression and T stage. Furthermore, higher LEA expression was also significantly associated with shorter OS in univariate and multivariate analyses, indicating that LEA is an independent indicator to predict poor prognosis in CRC.

An optimal biomarker with clinical efficiency should meet several requirements: first, high specificity, which ensures detection accuracy and avoids false positive; second, excellent sensitivity, which offers effective detection rate and avoids false negative; third, convenient detection access, such as liquid biopsy sampling, which facilitates clinical multiple sampling for early diagnosis and therapy monitoring. PODXL has been proven to have the potential for use in predicting metastasis, estimating prognosis and guiding medication.^38^, ^39^, ^40^, ^41^ Therefore, it is necessary for PODXL to have these advantages.

Current researches reported that PODXL can be glycosylated differentially in different tissues, possibly resulting in different functions.^42^ In podocyte of normal renal tissue, PODXL is 160‐165 kDa,^23^, ^24^, ^43^ while in cancer tissues, PODXL displays changeable molecular weight due to the diversity of post‐translational glycosylation.^21^ For example, in ovarian cancer, PODXL was found to have a molecular weight of 120‐150 kDa, which down‐regulated the cell adhesion,^37^ while in embryonal carcinoma, PODXL was detected to have a molecular weight of approximately 200 kDa due to the addition of keratin‐sulfated glycosaminoglycan.^44^ In this study, we identified a PODXL with a molecular weight of approximately 230 kDa in CL187 cells, which could serve as an indicator of CRC prognosis. Thus, it is expected that distinguishing and detecting the difference might enhance the specificity for PODXL detection. Currently, several commercial antibodies have been developed to recognize different sites of PODXL, such as mAbs 3D3 and 4F10 against PODXL core protein,^45^ and mAbs TRA‐1‐160 and R‐10G against the keratin sulfate‐related structures of PODXL.^46^ To verify the application potential of LEA, a PODXL recognized by mAb ND‐1 against its sialic acid residues, we comparatively analyzed the LEA/PODXL expression using 3D3 against PODXL and ND‐1. 3D3 is a widely used monoclonal antibody binding to amino acid residues 254‐415 of extracellular domain of the PODXL.^31^, ^47^ Among five CRC cell lines detected, four cell lines could be detected by 3D3 and three‐ones did by ND‐1 (Figure S3). Notably, by using ND‐1, Colo205 cells, derived from a CRC patient with Dukes’ type D,^48^ displayed a much higher LEA/PODXL expression than CL187 and LS174T cells from patients with Dukes’ type B,^49^ which is in agreement with the correlation of LEA/PODXL expression to CRC T stage (Table 1). Unfortunately, 3D3 failed to detect Colo205 cells in spite of achieving a higher positive detection rate than ND‐1, indicating that detection of LEA/PODXL using ND‐1 might have more excellent specificity relative to that using 3D3. Many reports showed that increased sialylation of proteins is associated with strong invasiveness and poor prognosis of cancer patients.^50^, ^51^ Moreover, several researches demonstrated that antibodies targeting sialic acid epitope had preferential specificity than that against core protein for biomarker detection.^52^ Currently, many antibodies targeting glycosyl epitope, such as CA19‐9, CA72‐4, and CEA, have been widely applied for clinical detection.

Our comparative study also demonstrated that ND‐1‐based detection toward LEA/PODXL had superior sensitivity to 3D3. When detected using ND‐1, Colo205 cells displayed a strong LEA/PODXL expression, while using 3D3, there was no detectable expression. The possible reason is that the LEA/PODXL epitope recognized by 3D3 was covered by glycocalyx due to its highly glycosylated modification.^53^ Further evaluation of LEA expression recognized by ND‐1 in CRC tissues by IHC also revealed higher imaging signal than that by 3D3, especially in metastatic tissues (Figure 5A a2).

Apart from superior specificity and sensitivity, noninvasive access ability would also facilitate effective application of biomarkers in clinical trials. Our previous study has detected the LEA in CRC patient serum and ascites.^15^ In addition, Fernández et al^54^ demonstrated that PODXL could be proteolytically cleaved on its extracellular domain and released into extracellular space. Recently, several studies found that PODXL could be secreted in exosome form.^55^, ^56^ Exosomes are small membrane vesicles ranging from 30 to 100 nm in size and play a critical role in intercellular communication by their various contents.^57^ There are increasing evidences that cancer‐derived exosomes may contribute to tumorigenesis and metastasis and served as a potential biomarker.^58^, ^59^ Meanwhile, due to their presence in most body fluids, exosomes might sever as noninvasive clinical biomarkers.^60^ Accordingly, we extracted exosomes from CL187 cell supernatant and CRC patient peripheral blood, and demonstrated that LEA/PODXL highly accumulated in the exosomes using ND‐1‐probed western blotting assay. To our best knowledge, it is the first time to find the LEA/PODXL expression in cancer cell‐derived exosomes. Currently, the effect of PODXL on cancer progression remains largely unknown; our finding therefore not only favors clinical detection of PODXL but also advances the understanding of PODXL effect on regulating cancer microenvironment.

In summary, the present study identified LEA as an approximately 230 kDa PODXL glycoprotein, and its terminal sialic acid is the essential component of the LEA epitope. Moreover, LEA functions as an independent predictor for the progression of CRC. Furthermore, ND‐1 showed superior sensitivity and specificity to commercial mAb 3D3, which recognizes PODXL core protein, in CRC samples, and LEA/PODXL can be secreted in exosomes derived from cancer cells and CRC patient plasma. All results suggest that LEA is a new potential biomarker, which might be used in noninvasive detection via exosome‐based bodily fluids of CRC patients.

## CONFLICT OF INTERESTS

None declared.
