# Supplementary figures and images for: Identification of LEA, a podocalyxin‐like glycoprotein, as a predictor for the progression of colorectal cancer
Source: Cancer Med. 2018 Sep 12;7(10):5155–66. doi: 10.1002/cam4.1765 (PMC6198229; doi:10.1002/cam4.1765)

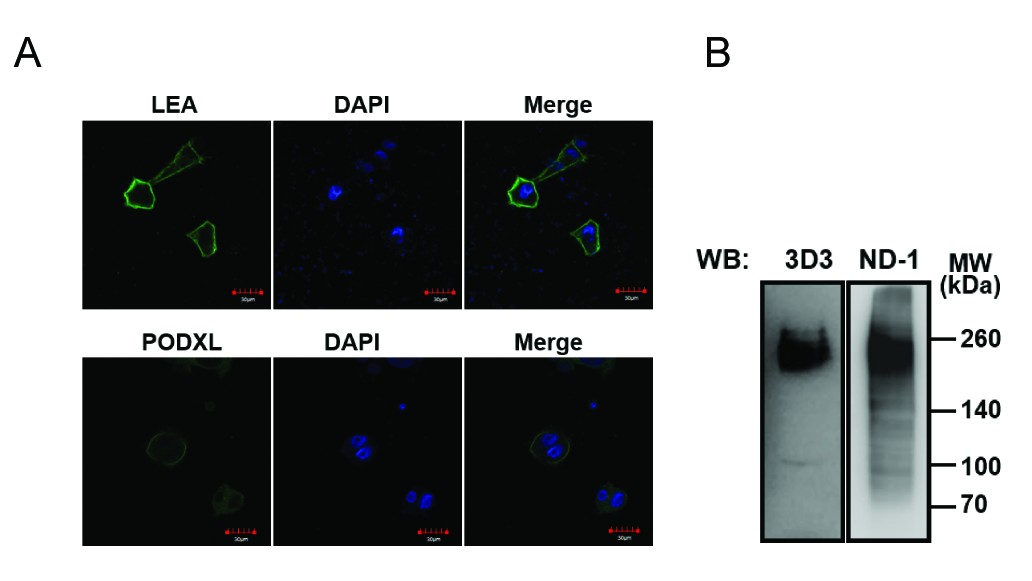

Supplement: Supplementary file 1 [file CAM4-7-5155-s001.tif]

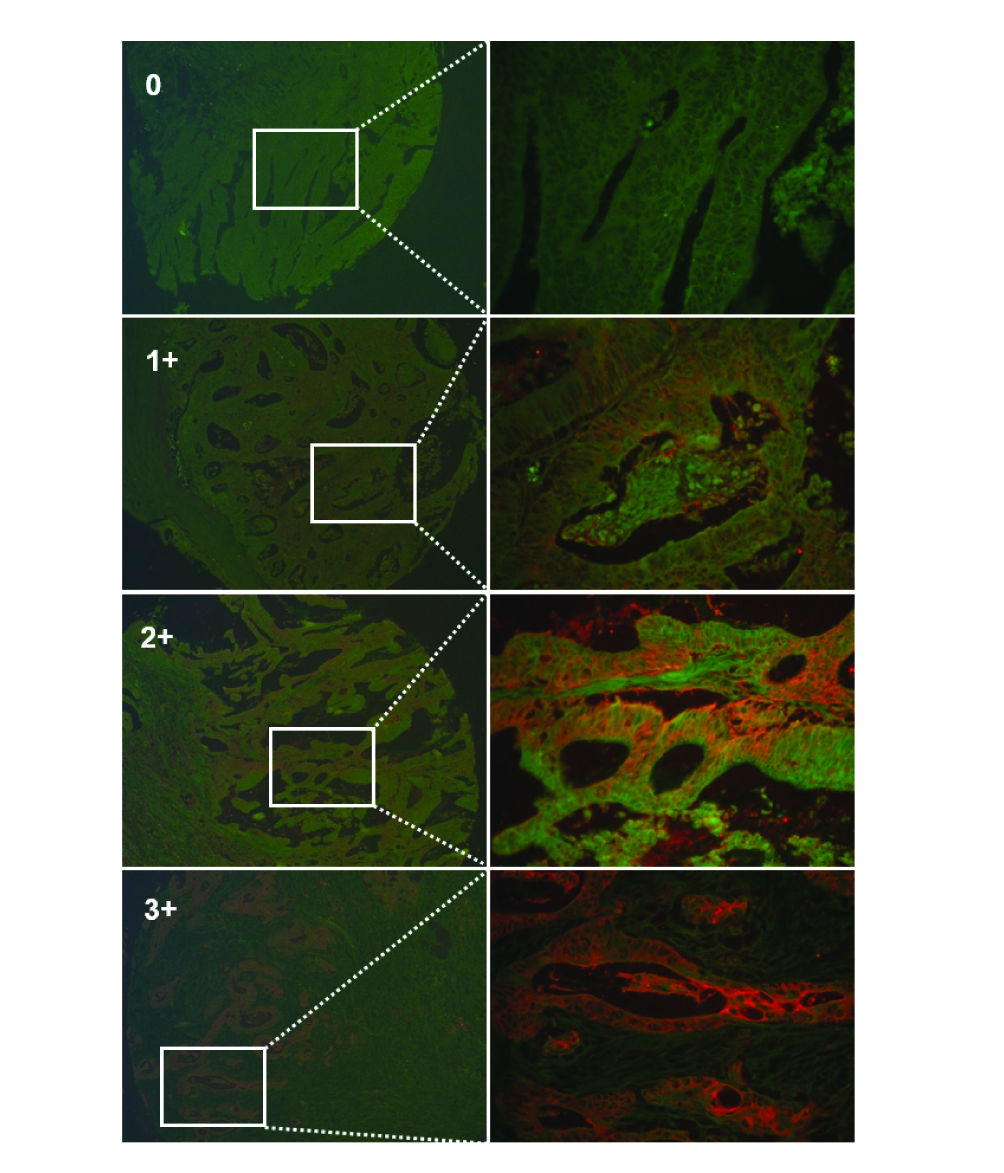

Supplement: Supplementary file 2 [file CAM4-7-5155-s002.tif]

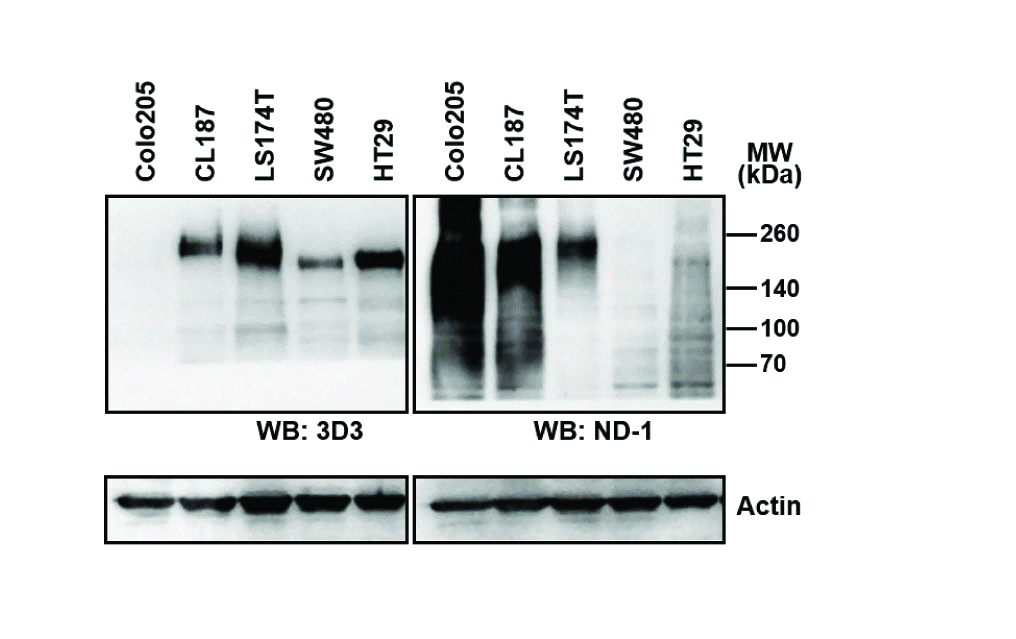

Supplement: Supplementary file 3 [file CAM4-7-5155-s003.tif]
