# Supplementary material for: Identification of LEA, a podocalyxin‐like glycoprotein, as a predictor for the progression of colorectal cancer
Source: Cancer Med. 2018 Sep 12;7(10):5155–66. doi: 10.1002/cam4.1765 (PMC6198229; doi:10.1002/cam4.1765)
Supplement: Supplementary file 4 [file CAM4-7-5155-s004.docx]

**Supporting information**

Supplementary Figure 1. Comparative analysis of LEA and PODXL in cellular localization by immunofluorescence assay (A) and molecular weight by western blotting (B). Scale bars: 30μm.

Supplementary Figure 2. Representative QD-IHC images based on LEA expression levels in CRC TMA. Images (×100, ×400 magnification) representing all different scores from 0 to 3+. Score 0 = negative staining, score 1+ = weak staining, score 2+ = moderate staining, and score 3+ = strong staining.

Supplementary Figure 3. Analysis of LEA and PODXL expression in five CRC cell lines by western blotting using ND-1 and 3D3 antibodies, respectively.
